# Supplementary material for: Barriers and Enablers of Health Service Utilisation for Childhood Skin Infections in Remote Aboriginal Communities of Western Australia
Source: Int J Environ Res Public Health. 2020 Jan 28;17(3):808. doi: 10.3390/ijerph17030808 (PMC7037003; doi:10.3390/ijerph17030808)
Supplement: Supplementary file 1 [file ijerph-17-00808-s001.pdf]

International Journal of
Environmental Research and Public Health

MDPI

Factors impacting on health service utilisation for skin infections in a remote region of Western Australia.

Supplementary material: a structured compilation of selected quotes

The quotes are organised according to the health service utilisation matrix and the various themes that were categorised in that matrix. The header included on each page shows which factor of the Andersen model (predisposing, enabling, need) and which level (client, provider, system) the quotes on the page are in relation to.

Within each theme, quotes are printed in the following order: parents/carers ('C'), healthcare practitioners ('HP'), other service provider ('OSP').

Names mentioned in quotes are aliases. Quotes are ascribed to one of the three participant groups (parents/carers, healthcare practitioners, other service providers) and not individuals for anonymity.

Quotes provide an overview of the range of attitudes and perceptions that were captured regarding the various themes documented in the health service utilisation matrix. They are reflections of an individual's opinion and do not represent the opinion of the authors. We have strived to provide a balanced and honest representation of the views expressed.

Unless specified otherwise at the start of the quote, quotes about health services are in reference to community Aboriginal medical services and staff.

Shyness & shame

(discussed by C, HP, OSP)

"People get shame with the sores. You know they don't like to show anyone they have them." (carer)

"[in reference to mainstream health services in town E] The main problem is the shame and judgment going to the clinic. […] The staff at the clinic think that Aboriginal people carry disease. They think 'oh not that black disease-carrying person again'. That's why the Aboriginal people don't want to go to the clinic." (carer)

"Nevertheless, there's a shame factor involved, and that will probably contribute to people not coming and seeking out help. In communities where there's a good relationship between the nurse they'll come at an early stage for treatment, and again in communities where there is no good relationship then people will not come even if it's uncomfortable perhaps." (healthcare practitioner)

"[comment in relation to skin sores] Shame is a big thing here too I think. I have heard the mums say that before." (other service provider)

"[in reference to children going to school in town E] Some of the kids I see, they won't leave the house because of the embarrassment of it. The sores. When they're really visible, they have them on their feet and around their hands and that, they don't want to go to school because the shame, and kids make fun of them." (other service provider)

fear of judgement, incl. DCP&FS (Department for Child Protection and Family Services)

(discussed by C, OSP)

"[in reference to mainstream health services in town E] All the health staff have so much judgement when we go into the clinic. They judge us and wonder why we haven't taken them [our children] in earlier." (carer)

"And then of course there's others that are still a bit weary because of the welfare issue. Just too scared to take their kids in because they are worried about the judgement and harshness from the doctors, welfare, things like that. […] That's their biggest fear. And I've tried to get across to them that if you take your child in when they are sick, they can see that you are trying to do the right thing by your kids. […] I said "If you turn around and you get in early, you won't have that problem, but if you get to that point then the kids get so sick you have to take them, it's a welfare issue, because you've neglected them, you haven't gotten them in soon.". I say the same thing to –and I was trying to explain to them- it's the same thing for me. I said "I got told off myself because my daughter's boil got so big", I said "I tried absolutely everything and then finally thought well, ok, I'll take her to the doctor's and the doctor told me off for letting it go for so long.". So it's a… I said "It's just the way it is." and I said… well, I guess up here it's just that bit more… they are very harsh with their speech. […] I spoke to a lot of the mums there. They all talk about Stolen Generation happening all over again. You take your kid to the doctor and before you know it they'll be knocking on your door the next day. And it's like… It's very sad." (other service provider)

**FACTOR:**	**predisposing**	enabling	need
**LEVEL:**	**client**	provider	system

traditional remedies & self-treatment

(discussed by C, HP, OSP)

"[commenting on the use of bush medicine for treating skin sores] We use those gum tree leaves. And we boil it and then put it on the sores in the bath and it makes them dry and it goes away." (carer)

"…the betadine cream when you put it on it dries and gets healed." (carer)

"Sometimes when they [skin sores] get worse and worse I get them the medicine soap." (carer)

"They smoke the houses and they use bush medicine. I mean, I've seen some horrendous wounds and boils that have had bush medicine on them, and they come in, and they nearly lost a foot or… Because they've not been… they've not come to the clinic. While a course of antibiotics may have prevented it. I say 'may', don't know. It's one of those things, you don't know. But the… White man's medicine and black fella stuff can work in conjunction. But they… I don't know… Some of the older people believe that older medicine is the way. And you can still talk to them. The ladies will go out and come back with bush medicine and stuff. And it seems to be that they only come into the clinic when it's not working." (healthcare practitioner)

"Yes. I do think that bush medicine is absolutely critical, and I think they should be working at tandem with the clinic to co-share, and that suggestion was also put in [name of town], because people were fearful of going off on the plane, and never coming back, and being taken off to hospital." (other service provider)

negative past experiences with clinic

(discussed by C, HP, OSP)

"The clinic doesn't ask us if we want a choice- they only give the needle. They don't ever give us the syrup." (carer)

"Some just make a dressing and don't do anything eh. They just say come back tomorrow and don't do anything." (carer)

"[in reference to mainstream health service in town E] The staff just gave me Panadol for my sores. Then it got really, really bad." (carer)

"I mean, these kids, alright… If you stick a needle in them… There's one little girl who's terrified… Comes in the clinic, she sees a white face, she bursts into tears. You know? She's had LA Bicillin three times. It hurts. It's not a pleasant thing to do. To have, let alone do. And you know when this kid looks at you, the first thing she thinks is "You're going to stick a needle in me.". You know? Straight away. So they're going to avoid coming. And as they get older, they're going "I'm not going there.", that's what's going to happen. So… yeah, LA Bicillin is a really good drug. Penicillin works wonders up here. You know… works wonders… Procaine… Wonderful drugs. But getting it into them… They know what you're going to do and… it's very difficult sometimes. And to hold a child down and inject a child… until you've done it, you don't understand… 'cause I've got kids… I've seen my children… Thankfully my kids have never had to have LA Bicillin or Procaine, you know. And I know how I would've felt… But… As these kids grow up, they only see themselves with these sores and they know a white fella is going to stick a needle in them because mum won't clean the sores and won't give them the medicine that she should." (healthcare practitioner)

**FACTOR:**	**predisposing**	enabling	need
**LEVEL:**	**client**	provider	system

"[in referring to mainstream health services in town E] I'm hoping she'll come back today for her boils, but… There was just no way of getting her any type of help. And she would not go to the hospital, because they give that… something bicillin injection, some sort of… you know what I'm talking about? "I don't want to go there, they give you a needle". We tried to get her in the car. She was ready to run even though she was sick as a dog. And I thought it's better for her to be here sick as a dog than running and something happening. So in the end I just… put her in bed and helped her through it. She was… She was really unwell. Seeing sick kids, but she was scary sick. If you saw her, it was ridiculous." (other service provider)

"I have brought a lady to the clinic before for a full body check-up. They wanted to do them, and they had-- I'd spoken to the nurse that I could round some people up and bring them in. That consisted of I spoke to the women and said what it involved, like hopping on the scales, measuring their waste, looking at eyes and ears, and asking some questions. I used my relationships with them to bring them to the clinic. We got there, and we were turned away. The nurse was busy. It was just wrong timing, and it wasn't a good experience for the person." (other service provider)

apathy & disempowerment

(discussed by HP, OSP)

"I think that on a scale of priority, health comes at the bottom simply because it's not anywhere different in any part of the world. If drug and alcohol issues, nutrition, family issues, domestic violence - all of those things - money issues, and grief, their position in the world, their place in our society, really that's-- most people out here would be confronted with personal life traumas as stress factors that most of us really only maybe experience every ten years or five years. But they'll do it in a year. A grandmother that has to look after four or five little kids, doesn't get any financial aid, hardly can feed herself, her health is down there somewhere in the bottom [of priorities]. There's a powerlessness there where I think that people are just-- there's a kind of a, not giving up, but resigned." (healthcare practitioner)

"We've had a couple of cases where these kids are really covered in stuff and they should be up at the clinic and they haven't been. It isn't really our responsibility to take them up there but do if we have to. If something happens at school like a cut or whatever, we fix that up and take them up there and that sort of stuff. Basically there's a bit of apathy around in terms of health." (other service provider)

priorities, values & norms – health & child rearing

(discussed by HP, OSP)

"The parents don't seem to have the same… And I can't generalise here. Some parents are wonderful with the kids. It tends to be the grandmothers more so that tend to be looking after the young fellas and young girls. But, there are families where the kids are just left." (healthcare practitioner)

"For them, it's priorities. This week there are two funerals and everyone is out of town. These are the barriers […] They prioritise things like holidays and funerals." (healthcare practitioner)

"Yeah, I can't articulate it as well as I'd like to, but it's that thing about that whole connection - the whole community raises the kids. To the outside world, that might sometimes look like neglect, but actually there is a

**FACTOR:**	**predisposing**	enabling	need
**LEVEL:**	**client**	provider	system

notion, an instilled sort of thought, that the whole community will care for the kids regardless of who they are and where they're wandering. […] Just that empowerment behind children being really independent and that's how they grow. And I guess, more than anything, is that spirituality. The Aboriginal way of being, that spirituality to their family, to their community, to themselves is quite special and that in an early childhood setting in a mainstream world, that gets missed" (other service provider)

"Because there's no word for tomorrow in Martu you can't explain what will happen. When you tell them that their runny nose and that the germ that does that is the germ that affects the kidneys and later on in life they'll be looking at kidney failure or renal dialysis's, they don't look that far ahead. It's today. It's for now." (other service provider)

"The parents only think about trying to feed their children they don't think about their sores. […] The structure of the family goes father then mother then children so treating children is the last priority." (other service provider)

cultural taboos

(discussed by HP, OSP)

"Also, understand from the cultural point of view how aboriginal people think and do things. Mostly they are not… They feel ashamed to be outspoken, or to be heard, or anything to link somehting about them to anyone, because of… community and skin group and family connection. Sometimes an Aboriginal person will come to a clinic, they'll find an in-law… and they will say "Oh, look, I'll come back later." – "No, no, no." If you don't understand [that reaction] and [insist]… "No, please, come in!". No, no, no. Just let them go. And then they might realise "Oh, that's an in-law in the other room. They knew he was in the clinic. So they wouldn't be able to speak". If you are not aware of that, you'll be like "Oh, what's wrong?"" (healthcare practitioner)

"With the strength of the tradition of the culture here though, someone from this community being the nurse would be inappropriate, they wouldn't be able to treat probably three or four skin groups, and they wouldn't be able to-- […] so all these familial relationships and skin group relationships, that's a huge factor in coordinating anything." (other service provider)

tolerance for discomfort & pain

(discussed by HP, OSP)

"Because, you know, it's being a sook to report pain. It's being a… Suck it up. Be a man. A man should not cry." Or such things. So… being tough… That's why you find even health checks are very low on men, for a simple reason: men don't go easily, you know. Woman should go, 'whinge' and you know… But men, no. They should suck it up. They [boys] should not even cry. If they fall down and stuff like that, let them suck it up." (healthcare practitioner)

"They're extremely painful [referring to skin sores] but these kids never, ever complain. You can be damn sure if a Martu kid comes to you and say, "Miss, I'm paining" they're in agony because they just don't complain. But I've seen kids with boils in here and on their knuckles so they can't move their hands, they can't hold a pen, they can't write. Kids with boils on their bums. […] You never get a Martu kid coming up and going, "Oh Miss, I've got a boil," like you do with the little white kids." (other service provider)

**FACTOR:**	**predisposing**	enabling	need
**LEVEL:**	**client**	provider	system

"I don't think they're ashamed of the sore itself. They're ashamed of feeling pain. Or maybe 'shame' isn't quite the right word, but it's not… You know, it's not something to be open about. Like, if you're a tough person, you don't feel pain. More that kind of attitude." (other service provider)

lacking (health) education
(discussed by HP, OSP)

"Well… I mean… Lack of education, of school early on. But you can only teach a child so much." (healthcare practitioner)

"And the education - even though the kids are here to get an education - but a lot of the families are uneducated" (other service provider)

"I think, again, it's education. It's not knowing that it's bad. I think many of them if they actually understood what it is and what it's all about will take far better care of their kids. But they just don't know. They're uninformed. So, that's the way it's always been." (other service provider)

language barrier

(discussed by C, OSP)

"[in reference to mainstream health service in town E] They [healthcare practitioners] also expect us to know what they're saying, like they talk down to us you know. They use all this doctor talk and I don't have a clue." (carer)

"90% of the nurses over there they don't understand Martu. They have got no idea what they're saying. I took a girl over to see a doctor and the doctor had been here 15 years and I had to translate for them. There is a big major language barrier. The staff won't break it down to simple language […] But the Martu people will say they understand. They will nod their head to the doctor and say they understand even though they don't." (other service provider)

FACTOR:	predisposing	enabling	need
LEVEL:	client	provider	system

**FACTOR:**	**predisposing**	enabling	need
**LEVEL:**	client	**provider**	system

Prejudice

(discussed by C)

"[in reference to mainstream health services in town E] All the health staff have so much judgment when we go into the clinic. They judge us and wonder why we haven't taken them [our children] in earlier." (carer)

"[in reference to mainstream health services in town E] The main problem is the shame and judgment going to the clinic. […] The staff at the clinic think that Aboriginal people carry disease. They think 'oh not that black disease-carrying person again'. That's why the Aboriginal people don't want to go to the clinic." (carer)

"[in reference to mainstream health services in town E] The staff at the clinic are racist. The mums want an Aboriginal worker there not a white person." (carer)

Stressors associated with work environment

(discussed by HP, OSP)

"Challenging… not because of being busy, but because of remoteness, loneliness and isolation. The isolation there really is heavy because… basically you know the next doctor for your support is 900km away. The next community is [name of other community]. It's also in the middle of nowhere. So basically you are very far, you really feel it, you're very far, there's nothing, very few people to talk to. And during the festive season, like now, everyone goes over heat. It reaches up to 55 degrees there. They leave the community. So sometimes you are only two [people] in the community." (healthcare practitioner)

"A lot of pressure on the nurses. On call. Long long hours. I did a… 11 o'clock in the morning, to 2:20am the following day shift. We're supposed to be paid… The remuneration we get is supposed to cover the hours that we do on call. No way, no way does it match up to the amount of hours that you obviously do for what we get. That causes a lot of consternation among the nurses, because… We've all got a life. I don't live there to work 24/7. I'm not… Albert Schweitzer. You know. I go out there for a reason. I have a partner, I have a mortgage." (healthcare practitioner)

"Then again the upside or downside is that the mob think the nurse is there for 24 hours a day, and they'll knock on the door at 11:00 at night for a headache tablet, which they've had all day, and you can understand why nurses would get pretty pissed off. Yeah go away, come back tomorrow morning. I'm closed from 12:00 till 1:00, it's my lunchtime, come at 11:30 not 12:30. But the mob don't have any concept of time. I've got a problem then I have to go and fix it, now. Preferably yesterday. That's just the mob. We don't see that at the school, because we're not being hassled, if you want to call it hassling. But as a clinic, I suppose they have to make a call whether it's life threatening or not." (other service providers)

jadedness/frustration

(discussed by HP)

**FACTOR:**	**predisposing**	enabling	need
**LEVEL:**	**client**	provider	system

**FACTOR:**	**predisposing**	enabling	need
**LEVEL:**	client	**provider**	system

"It's very difficult, because you try to educate people in diet, and smoking and grog. But they're all things that they just… do what the hell they want. They smoke, they drink. When they come into town, they binge, and they eat crap food really. They don't eat good food. And I… Sometimes when I… The amount of time and effort that I put into actually educating the people on what they should eat, it just seems to go straight over their head." (healthcare practitioner)

"It's very difficult, when you're… It's like, you start something, you get it right and then… A month later it's back again. It's just… They've not learnt the reasons behind why they're becoming ill. And I'm a big… You know… What's the word I'm looking for now? I… I believe in ownership. I won't spoon-feed these people. You can only do so much for them, and ownership of their illness is something they have to do. I couldn't go to my doctor and say "I want this, this and this.", and they'd go "Right.". They would say in the end to me: "Mr. [surname of interviewee], you can't come again. We're getting nowhere here. You have got to take ownership of what's wrong with you." (healthcare practitioner)

training/knowledge gaps

(discussed by HP, OSP)

"Yeah, yeah. Because none of us were really given that much cultural… sort of understanding, before… up until about a year, but I was given nothing… that was a huge, huge shock for me.
" (healthcare practitioner)

"The health staff are learning as they go. It is not any disrespect to the doctors or nurses […] It's a difficult injection to give [in reference to BPG] but it depends on the nurse giving it. It just depends on your technique and training. Some of them are pretty rough up there […] The nurses don't do the 12 months additional training where you learn the culture, learn the ways. And that is important. This is going to become un-stuck soon because they don't know who is allowed in the clinic." (service provider)

**FACTOR:**	**predisposing**	enabling	need
**LEVEL:**	**client**	provider	system

**FACTOR:**	**predisposing**	enabling	need
**LEVEL:**	client	provider	**system**

reconciling traditional and modern medicine

(discussed by HP, OSP)

"In some communities where there's a lot of strong elder people that still practise Maban, or bush medicine, I can almost guarantee you that they would've seen a Maban before they've seen me, or it's part and parcel. They coexist, and we don't acknowledge that enough. I know that in some other parts of Australia, it's starting to involve it more. But in these parts here, I find that almost unbelievable that as an Aboriginal health organisation we are not encouraging Aboriginal healing side by side in our practice. Because it's happening anyway. I know they're highly protective of it, so it's not just because we don't acknowledge it. […] I think that there is another avenue for us to maintain their culture by encouraging that, so young people feel they want to go down that way. I mean, it happens irrespective of us, not that we're training people up for that. Maybe it's happening in a healthy way? I certainly worked with people on that level, and have let the people use the clinic for use of Maban, and I've seen it practised in front of me. And that again comes down to whether they trust you. Whether I believe in it or not is irrelevant. If you really want to be effective in your practices then you also have to acknowledge that that part also plays a part whether it's placebo or does have an effect." (healthcare practitioner)

"One time I was called into a home. This woman was really sick, but not sick as an emergency, So… I go there. After reaching there, they say "Look, she's been bewitched. By so and so. In [name of community], yeah. She's been bewitched. So we've brought our medicine man." Just doing stuff. "Once he's finished then you can do your stuff." Basically she had infection. So she had infection. She had all these sores which she did not treat, and then she got a fever and stuff. She was dehydrated. The first thing she needed was rehydration, put on a drip and stuff. From the medical point of view I'm like "Oh, look you guys." -sunken eyes and that- "I know what to do. But because you've said she's been bewitched, and you are doing something…". I thought "30 minutes of them doing that will not change anything really.", so I waited. I sat there. Waited. He was there, doing his stuff. I was like "OK. I will just sit here and wait, and once he's finished, then I'll take her to the clinic.". So I sat there. And then I went with the… You know, another nurse "Oh, that's bullshit, common man! Just tell them that's bullshit, then we…". I'm like "No no no no.". Respect their view and respect… You know. So look, it's that kind of respect which they really need and when you do it that way, you'll find that you'll gain their cooperation. Don't say "No, me I know it all.". You know from the scientific point of view, they know from the spiritual side, you can't argue with their spiritual issues. Those are entrenched beliefs of health from the time… So you are not there really to change that. And then after that we got the patient, rehydrated her, pumped her iv, she was ok. But, you know, the next day when I met them "You see, now she's ok. The JuJu man did it.". But me, clinically I know that, look… But "Yeah yeah yeah." You know?" (healthcare practitioner)

"We had a serious problem on one night - was it Friday - when one child came here and said "Oh no, there was the monster last night at this woman's house and it attacked the old lady and--". They are still very much into these spiritual and the supernatural and that stuff. She thought the old lady got attacked; there an attack. She actually came back here in the evening and she was like-- and we get her on the plane and so on and so. We still haven't got the full information, but it was a medical condition, a stroke, or whatever the case may be." (other service provider)

**FACTOR:**	**predisposing**	enabling	need
**LEVEL:**	**client**	provider	system

**FACTOR:**	predisposing	**enabling**	need
**LEVEL:**	**client**	provider	system

low costs associated with medical care

(discussed by C, HP, OSP)

"I usually go to [name of community] [from town E] to get the cream because I can get it from the clinic for free." (carer)

"[in reference to mainstream health services in town E] My son had three appointments in Perth but they didn't want to pay for their trip and I can't afford that much." (carer)

"Thank God they don't have to pay! If they had to pay to see a doctor here, by Christ, you'd have such a high mortality rate, or people losing limbs, if they had to go pay a doctor. You know. It just wouldn't happen." (healthcare practitioner)

"[in reference to mainstream health services in town E] That's the other problem, it is expensive to see a doctor in this town. It's $90 for adults, it's free for children. […] My husband's Aboriginal, he still has to pay the $90, unless he can present a healthcare card or something like that. And a lot of the Aboriginal people don't carry their healthcare cards on them, so it's pay up and then you get to see a doctor. And that's why… 'I don't have the money'" (other service provider)

access to 'Closing the Gap' benefits

(discussed by C, HP)

"[in reference to mainstream health services in town E] They don't put the 'Closing the Gap' anymore on the script. If you ask the doctor to write 'CTG' on the script, they will say 'nah we don't do that anymore.' They just think all the black fellas are stupid and are trying to rob you." (carer)

"[in reference to mainstream health services in town E] Even here they do this closing the gap scheme, but all of the black fellas they don't know that they can go there and see the doctor for free. They need to tell us this stuff you know?" (carer)

"If they have 'closing the gap' benefits the consultations are free. But it's up to the doctors to write 'CTG' on the script otherwise they have to pay full price. Many of the times the doctors don't write it on there for them because they don't even know." (healthcare practitioner)

good perception of clinic staff

(discussed by C, HP, OSP)

"[in reference to mainstream health services in town E] You can sit there for 3 hours before you even see a doctor. They don't even look like they're busy- they are pushing pens and just yarning. They're just lazy." (carer)

"[in reference to mainstream health services in town E] The doctors here can't wait to get rid of you quick enough." (carer)

**FACTOR:**	**predisposing**	enabling	need
**LEVEL:**	**client**	provider	system

FACTOR:	predisposing	enabling	need
LEVEL:	client	provider	system

"[in reference to mainstream health services in town E] We have one regular doctor here. The rest of them are FIFOs [fly-in fly-out] and they're dickheads." (carer)

"I used to go to [Name of former long-term health worker] everyday when he was here and he knew always what to do, but the new staff there at the clinic don't know anything they just look it up in a book. I don't go to there anymore." (carer)

"Another component to it, why things may not be as good, I think if there's a good relationship between nursing staff or medical staff in the community, parents are more likely to bring their children, or the children come themselves at an early stage. And then also depends on how school and the health centres work together. If there's a good working relationship and the community feels good about the health staff, they tend to come early. If they don't then it's left until the last moment." (healthcare practitioner)

"[in reference to AMS community clinic] I really can't speak for them because every time I go to the clinic I'm fixed up well and there's no hassle there. They're all pretty concerned and pretty on the ball, so I can't see that they wouldn't do the same thing for the community people that go up there. I've been up and sat there when people have been in the waiting room. They get shown through and given medicine and stuff so to me that works as well as it can. But I think it's a matter of getting to the care, is part of the problem. You know what I mean?" (other service provider)

health status of carer

(discussed by OSP)

"A lot of these people who are looking after these kids have early stage dementia or other major health problems themselves." (service provider)

FACTOR:	predisposing	enabling	need
LEVEL:	client	provider	system

**FACTOR:**	predisposing	**enabling**	need
**LEVEL:**	client	**provider**	system

engaging & culturally secure staff & practices

(discussed by C, HP, OSP)

"[in reference to mainstream health services in town E] Even the old doctor here in [name of town E], he has been here for 30 years but he doesn't have a rapport with the community. He doesn't have a really good personality, it took nearly 3 years to get a smile off him!" (carer)

"[in reference to mainstream health services in town E] We should have Aboriginal health workers because they will make sure everything is right because they have had so much primary health care." (carer)

"[in reference to mainstream health services in town E] The staff at the clinic are racist. The mums want an Aboriginal worker there not a white person." (carer)

"I think just being friendly [chuckles], and genuine, and actually as I've said make kids welcome. Make them feel like the clinic is a place where you come to get help. I find that here in particular, kids will come on their own and show me their sores. Often their sores or their cuts or whatever. And they engage. I engage them in how I interact with them, and I find that they're almost like happy to be coming and showing me their sore." (healthcare practitioner)

"I think that's all the attitude from whoever is in the clinic makes all the difference. People need to feel comfortable to come to the clinic, and no issue is a small issue. They need to feel comfortable, they need to feel like you care about them. "We need you to take your tablets, because we don't want you to get sick. [...] They've got to feel comfortable coming to the clinic, and I mean we can be taken very much advantage of and you can get just absolutely totally worn out. There has to be some boundaries, but I want people to know that they can bring their kids up, and I don't care what time it is, if their kid's sick, I want them to bring their kid up to the clinic because I don't want to be flying that kid out because they've left it so long." (healthcare practitioner)

"[in reference to mainstream health services in town E] The doctors that are there are… I… I've met up with a Jew doctor when I had to take one of my boys in. I had an African doctor that didn't have a damn clue what he was talking about. I don't know how a UTI can be an ear infection, but hey, he knew it all more than me! And I've come across a Muslim doctor that just, you know, think they're just dirty people and that it's pretty much their own fault that they are like that. Even though they don't put it that way, you can see it in how the carry on and the way they talk. And out in the community, when you see them walk around and how they… Yeah… So, it's very sad, the doctors don't have it. And they… Some of them come straight from their country to here. One of the doctors that I got to know a little was Papa New Guinean. He was really getting the point across that in Papua New Guinea, there is no CentreLink. There is no… hand-out money, there is none of this, none of that. And he is "These people get more. There's money thrown at them and there's money they get off of the government every week and this money and that money. And look how they're living, it's their own fault that they are pretty much living like this. Back home we worked hard for our money, we worked hard to make sure our kids were well, we looked after our families.". And he just… that attitude of having a dig, you know." (other service provider)

established relationship between staff & client

(discussed by C, HP, OSP)

**FACTOR:**	**predisposing**	enabling	need
**LEVEL:**	**client**	provider	system

FACTOR:	predisposing	**enabling**	need
**LEVEL:**	client	**provider**	system

"The nurses come and go all the time [in reference to community AMS]. I'm not gonna see someone I don't know." (carer)

"He [in reference to former longterm healthcare practitioner] has my own blood. He know my boys when they was baby. The ones now, don't." (carer)

"[in reference to mainstream health services in town E] This midwife once was really polite and wrapped my baby up. But you go back the next day later, and they're gone." (carer)

"Nevertheless, there's a shame factor involved, and that will probably contribute to people not coming and seeking out help. In communities where there's a good relationship between the nurse they'll come at an early stage for treatment, and again in communities where there is no good relationship then people will not come even if it's uncomfortable perhaps." (healthcare practitioner)

"I have to say in my previous experience, rarely [referring to a strong relationship between clinic staff and clients]. There's been a few times where I've seen it, but rarely just because the turn-over of staff is so high really in those posts. There's generally just one nurse out on their own and they have more than enough-- to be on call for 12 hours a day. The turn-over, I guess, has meant that it's been difficult for people to maintain those kinds of relationships with the community for an extended period of time." (healthcare practitioner)

"In my own experience, and previous experience, families won't engage with those sorts of services unless there are people that they recognize, or people that they've had a relationship with for a long time. That's why Aboriginal health workers are so important." (other service provider)

"[Q: Trust in the clinic staff is an important issue?] Very much. Especially with the women. If there's a male doctor there, very much. That's something that has been a huge loss. It will be a huge loss to the community in the health side of things because [name of past health worker] has been here for 20 odd years, so he knew everybody, knew their history, delivered probably most of them. All of that sort of stuff, known them from children. That's a huge loss." [other

clinic waiting time not too long

(discussed by C, HP, OSP)

"I think they only have 1 or 2 nurses there and you gotta wait a long time." (carer)

"You can wait an hour and a half, two hours here." (carer)

"…you got other families in front of us. And there are probably only 2 nurses." (carer)

"[in reference to mainstream health services in town E] There is a long wait at the hospital. One time I had a boil on my leg and I got up at 4 o clock in the morning and went to the hospital and wait long time long time [and gave up] then I went home." (carer)

"I think a lot of parents don't come because they know sometimes there is a wait. Because there's only [name of nurse] and I at the moment." (healthcare practitioner)

FACTOR:	**predisposing**	enabling	need
**LEVEL:**	**client**	provider	system

**FACTOR:**	predisposing	**enabling**	need
**LEVEL:**	client	**provider**	system

"I think the waiting time, perhaps, puts a lot of people off. It puts me off [chuckles]. Oh, God. Hours. Even when it's quiet, it feels like you're there for hours at times." (other service provider)

acceptability of treatment

(discussed by C, HP, OSP)

"Sometimes the parents get hurtful for that kid having the needle. They don't want to see that kid screaming there from that needle. And that needle is big it's not a little needle." (carer)

"My little one won't drink the medicine. She doesn't like it. They gave her the drink medicine but she won't take it." (carer)

"It's really sad [giving the injection]. Really emotional seeing the mums. One child had an injection today and the mother was tearing up like she was about to cry." (healthcare practitioner)

"I mean, these kids, alright… If you stick a needle in them… There's one little girl who's terrified… Comes in the clinic, she sees a white face, she bursts into tears. You know? She's had LA Bicillin three times. It hurts. It's not a pleasant thing to do. To have, let alone do. And you know when this kid looks at you, the first thing she thinks is "You're going to stick a needle in me.". You know? Straight away. So they're going to avoid coming. And as they get older, they're going "I'm not going there.", that's what's going to happen. So… yeah, LA Bicillin is a really good drug. Penicillin works wonders up here. You know… works wonders… Procaine… Wonderful drugs. But getting it into them… They know what you're going to do and… it's very difficult sometimes. And to hold a child down and inject a child… until you've done it, you don't understand… 'cause I've got kids… I've seen my children… Thankfully my kids have never had to have LA Bicillin or Procaine, you know. And I know how I would've felt… But… As these kids grow up, they only see themselves with these sores and they know a white fella is going to stick a needle in them because mum won't clean the sores and won't give them the medicine that she should." (healthcare practitioner)

outreach activities

(discussed by C, OSP)

"They used to have people drive around to your house to remind you of your appointment. It's a duty of care thing. And would drop all the old guys and the chronic guys off at their appointment. They should do that again." (carer)

"Two years ago there was a nurse called Danielle and she was my aid of gold, and she would come up to the school and she would make sure everyone was dewormed. Every day, three days in a row, you got a little medicine on a powder pop stick and all of the kids got dewormed, and that was good. That really sorted out bottoms. She also would give the school cream, little things like that. The kids would rub it on to their hands and face, and it would soothen their skin. She'd also come up-- we had a band on the oval-- pretty much the whole community was there. All the old people. All the parents. She walked around giving everyone flu injections. In those times I saw massive amounts of collaborations." (other service provider)

**FACTOR:**	**predisposing**	enabling	need
**LEVEL:**	**client**	provider	system

FACTOR:	predisposing	**enabling**	need
**LEVEL:**	client	**provider**	system

"I have not seen them come over to the school. It's us going over to them. It's the school staff being proactive. In terms of-- at this stage I have not seen the clinic supporting the school with a range of health initiatives. […] I would like to see it being two way. It's great that they're more than willing to see any of our students at any time, however in terms of promoting preventative health, it would be great to see a bit of interaction going on there." (other service provider)

"We had a great nurse there 12 months ago that started with a worming program at the school. She used to come every three months and worm all the kids, and check their general health and well-being, and that sort of stuff, and it was fabulous. And then, she left and nobody's done it since." (other service provider)

clear communication re visiting health services
(discussed by C, OSP)

"The clinic staff don't let the mothers know when the baby doctors are coming." (carer)

"At least if the doctor comes, put pamphlets in everybody's mailbox. And just tell us 'she's coming', 'he's coming'" (carer)

"It would be good if they could notify the school or playgroup when they are coming so we can spread the word. […] They just don't inform the mums when the doctors are coming into town, therefore they have no idea. We need to advertise it on the newsletter board." (other service provider)

patient engagement

(discussed by C, OSP)

"Some just make a dressing and don't do anything eh. They just say come back tomorrow and don't do anything." (carer)

"[in reference to mainstream health services in town E] They also expect us to know what they're saying, like they talk down to us you know. They use all this doctor talk and I don't have a clue." (carer)

"[in reference to mainstream health services in town E] They don't even think about what they give, they just give something out without telling us what's wrong with us." (carer)

"The clinic doesn't ask us if we want a choice- they only give the needle. They don't ever give us the syrup." (carer)

"The health clinic doesn't give them an option for treatment for their skin but it's whatever the nurse on at the time wants. They don't care. And that's the problem." (other service provider)

"The staff don't even tell them what to do with the medication or how to get rid of the sores. They need to educate them you know? […] The health clinic doesn't even give them soap or any stuff to rub on the sores they only give them medicine and no other treatment – no information on it either." (other service provider)

comfortable clinic facility
(discussed by C, OSP)

FACTOR:	**predisposing**	enabling	need
**LEVEL:**	**client**	provider	system

**FACTOR:**	predisposing	**enabling**	need
**LEVEL:**	client	**provider**	system

"[in reference to mainstream health services in town E] I hate the clinic. It's so bad I don't even like going there sometimes." (carer)

"[in reference to mainstream health services in town E] Even the hospital here, it's dirty and old. It needs to be burnt down and rebuilt." (carer)

"What they love to see is heaps of photos of themselves [chuckles]. In the clinic. They love it, and they make it relevant to people here and what it means. […] and that's why I've always-- one of the things that I've put to my bosses is that I want computers here [in the clinic], and a better internet system so we can have adult literacy classes." (healthcare practitioner)

"But then I've been to the clinic, as I said, when I had this four-day bug, I had to go a few times. They love-- the people just sit there. Whether it's a community thing as well, you know, community meeting place that they can sit and they can get checked out, and they don't mind the advice taking, the medications that are offered to them. They quite like, but as far as maybe teaching them, "Well, here's some cloths and things; keep your sores clean," and what have you, I don't know how far it goes." (other service provider)

"I think something needs to happen over that clinic. To get them a bit more organised. The place is filthy, it needs to be cleaned up. The likelihood is they don't have a cleaner, because I know it's very hard to get a cleaner in remote communities. So those are the two things that stand out, and inter-agency communication, absolutely." (other service provider)

**FACTOR:**	**predisposing**	enabling	need
**LEVEL:**	**client**	provider	system

**FACTOR:**	predisposing	**enabling**	need
**LEVEL:**	client	provider	**system**

free or low-cost medical care

(discussed by HP, OSP)

"Thank God they don't have to pay! If they had to pay to see a doctor here, by Christ, you'd have such a high mortality rate, or people losing limbs, if they had to go pay a doctor. You know. It just wouldn't happen." (healthcare practitioner)

"[in reference to the utilisation of mainstream health services] Free for children, under a certain age. I can't remember how low it is, but it's free for the children." (other service provider)

ensuring adequate medical supplies

(discussed by C, HP)

"[in reference to mainstream health service in town E] Back at that clinic they gave me a script to go buy my son his own shot [LA Bicillin needle] but I shouldn't be buying my own because they should be provided from the hospital because every kid needs them." (carer)

"[Q: Do you find that there are any challenges there in terms of administering those treatments which are advised by the CARPA manual for various skin infections? For example, for skin sores?] They have to be in the medication room. [Q: So is that not always the case?] No. [Q: So is that a problem that you face regularly in remote clinics?] Yes, because whoever's working-- the first thing that I do in the clinic is go through the treatment manual and make sure that whatever's in the-- two things. Do people have any Webster Packs, and what's the drugs that are in here. That's the first thing that should be in your pharmacy. So then you can treat as per-- and your butt's covered as well. [Q: But that's not always the case?] No." (healthcare practitioner)

"Clinics that has a huge turnover of staff often runs out of stuff on a regular basis because the relieving staff don't know what to order. They don't know the amount of things to order. If they order, they might order one or two or three and then the clinic uses five a week. So it's a huge problem that the clinics aren't well enough equipped to have everything they need at any given time, but on the other side we can't have thousands of dollars in stock that we're using once a year." (healthcare practitioner)

adequate staff levels & low turnover

(discussed by HP, OSP)

"I think for the community it would be the consistency of the nurse. Like it's good to have people long-term and that often doesn't happen, you know." (healthcare practitioner)

"Well, the nurses always say out here that they're run off their feet, and it'd be obviously always understaffed. (other service provider)

"We have a lot of turnover at the clinic it's just so hard for the community to trust whoever is in the clinic because they don't know who they are going to see next." (other service provider)

**FACTOR:**	**predisposing**	enabling	need
**LEVEL:**	**client**	provider	system

**FACTOR:**	predisposing	**enabling**	need
**LEVEL:**	client	provider	**system**

"They have so many people going in [to the clinic] they actually don't have the time to talk or impart knowledge- they just treat." (other service provider)

"Well I think the thing is… You can't expect too much of the clinic, because they are absolutely under siege. If you've got one full-time nurse there… They're saviours here now… So then… I mean all these other things that sort of appear to be extras, like making contact with the school, or coming and delivering health programs or whatever…" (other service provider)

AMS governance & stability

(discussed by HP, OSP)

"I suppose when you look at a RAN [remote area nurse] point-of-view as well, you know, with their contractual basis and… I know [name of AMS] has been tumultuous, to say the least, over the last… you know… 10 years, by the sounds of it. As far as… you know… management coming and going." (healthcare practitioner)

"So [name of AMS] have had a few issues as well the last few years. I wouldn't say they're the most stable organisation, and that's probably impacted on their services as well. But I don't-- I can't really comment on that, I can only make observations. I don't really know the nitty gritty. But you can imagine if the organisation's in turmoil that would impact on their services." (other service provider)

efficient use of resources

(discussed by HP, OSP)

"What I find is that things are more successful when the health practitioner lives in the community. I find that even from my perspective, fly in, fly out - I call it the roadshow. Balloons and colouring stuff, and here's a hand, and here's this, and here's a chart, and blah blah, and disappear, don't work. I just can't see how a lot of the health promotion stuff that is regurgitated has any effect whatsoever. With all that money, I do believe again that our main thrust should be in the more mental and emotional sphere. That is one of the biggest barriers why people are disillusioned, people are depressed, people are suffering." (healthcare practitioner)

"All the ambulances have to be state-of-the-art… And how many are going to… When you look at statistics… Three per year, cardiac arrests. And you spend so much money to make this investment, and then only have three patients per year? And even if you do that anyway, where we are here, it's so far, if it's a major cardiac infarction, the patient will die anyway. So how about the downstream philosophy where you try to put a lot of resources there." (healthcare practitioner)

"Well, one thing I think it doesn't need is, you just throw money at things. And throwing money at it doesn't work. It has to be put in the right places. The money has to be made to work." (other service providers)

"A classic example on Friday, Mental Health were here, no idea they were coming. $10,000 for the plane and we've come to say hello. To me, give us $10,000, that is just a waste-- you feel like writing to the newspaper because that is criminal. How can you spend $10,000 to come and tell us who you are, and the people you want to see are not even here. So what's wrong with a phone call? But because we have this thing called tick a box,

**FACTOR:**	**predisposing**	enabling	need
**LEVEL:**	**client**	provider	system

**FACTOR:**	predisposing	**enabling**	need
**LEVEL:**	client	provider	**system**

where they have to say they've visited a community, regardless of whether they do anything or not, they say we visited." […] But I guess the real frustration for us is when you see a $10,000 plane and five or six people's wages, they come for one day, and you just think "That money could be so much better spent." (other service provider)

trained Aboriginal health workers

(discussed by C, HP, OSP)

"[in reference to mainstream health services in town E] We should have Aboriginal health workers because they will make sure everything is right because they have had so much primary health care." (carer)

"[in reference to mainstream health services in town E] …yeah but it's a shame, all the staff are pretty white. Or African. Hardly any Aboriginal." (carer)

"Clinics with Aboriginal health workers are often easier to get people back because you can send the health worker out to remind people. But in clinics without the health worker, you're sort of missing the link between the white person in the clinic and the indigenous people outside the clinic. I've been to clinics with no health worker and it's a lot harder to chase people to get them to come to the clinic for continuing treatment. But if you have a health worker, it's easier because they can go out and find the person and explain in their way that you need to come back and have your medicine. […] Local knowledge. They know who the people are. They know who to chase. They will also be able to tell me if people are here or not, if there's someone I need to catch for one reason or another. And it's a nice link, and they will tell you what's culturally appropriate and not appropriate. So having a good health worker is important for the nurses, and I think if you have a clinic with high turnover of nursing staff, having a health worker as your local resource is fantastic." (healthcare practitioner)

"Do I see Aboriginal health workers? Yes. Do I see long-term Aboriginal health workers? No. And that's for various reasons, particularly because they're put in a position of authority where they have to necessarily say no to their family sometimes which is culturally a big thing to be able to do." (other service provider)

good collaboration with other services

(discussed by HP, OSP)

"I know for some people it's not important, but I think it's very very important. Creating a positive environment between [name of AMS] and the community. Improving communication. Ok? Because where communication is poor, the community doesn't know what is happening, the thing is more stability. The community has got… a propensity to fight an organisation… and you'll be gone in seconds. You need cooperation. All these things we're talking about is cooperation. Without cooperation, you're wasting your time." (healthcare practitioner)

"And also if it's a good working relationship between the teachers and the nurses. Sometimes it doesn't always work well, and then there's reluctance to actually go and engage with each other because of communication issues, then I believe that only the community members suffer." (healthcare practitioner)

"The problem is, I think, the clinic doesn't talk that much with [name of community coordinator], the clinic doesn't talk that much with us. So they're very much isolated. […] But you can probably gather that communication is the key, and that's what's lacking." (other service provider)

**FACTOR:**	**predisposing**	enabling	need
**LEVEL:**	**client**	provider	system

**FACTOR:**	predisposing	**enabling**	need
**LEVEL:**	client	provider	**system**

community engagement & outreach policies

(discussed by HP, OSP)

"They no longer have any health staff coming to the school which they used to- they used to do health checks and stuff at school." (healthcare practitioner)

"One, approaching the community before you actually start, and finding out things that are appropriate and things that aren't appropriate. Have a practice run with the delivery, with appropriate people, and get feedback from that. Not just develop something and do it. It's got to be really well-thought out. It's building relationships with the community." (other service providers)

"There has been collaboration with the clinic, but my sense is that the clinic is so overwhelmed that they're not proactive in outreach. Any of the outreach has been instigated from the school." (other service provider)

"Yeah, I have. It's just I feel sometimes we could probably do a bit more for these kids than what's going. And maybe that's a [name of AMS] directive, I don't know. [name of AMS] have maybe said to nurses staying at clinics to let the problems come to you rather than go out and look for problems. And you can understand it from a nurse that they always seem to be overworked, why go looking for more work? But my argument is that sometimes kids don't know they've got a problem." (other service provider)

**FACTOR:**	**predisposing**	enabling	need
**LEVEL:**	**client**	provider	system

**FACTOR:**	predisposing	enabling	**need**
**LEVEL:**	**client**	provider	system

lacking awareness re skin infections

(discussed by C, HP, OSP)

"... she [her daughter] had a sore on her arm so I took her on a plane to Hedland [...] It looked like a blister it was black. I didn't know it was any problem. The baby doctor said I had to go." (carer)

"A lot of black fellas leave it until the end stages cos they aren't educated." (carer)

"They also normalise it. They think 'oh it will get better' they will only take the kids to the clinic if it's really, really bad" (carer)

"The mothers don't think it's an issue. Sometimes they will think 'oh that will be fine tomorrow', But they don't know what will develop behind this. In the blood stream you know?" (healthcare practitioner)

"It's not neglect, it's not laziness, it's just too vague. No one knows what it [the skin conditions] is." (other service provider)

"I think they need to educate and understand skin infections more, otherwise they become normalised and people don't bother to go until it's really bad." (other service provider)

delayed presentations & self-treatment

(discussed by C, HP, OSP)

"A lot of black fellas leave it until the end stages cos they aren't educated." (carer)

"Most people leave them until they get really, really bad and worse." (carer)

"I don't go to the doctor unless it's serious [in reference to skin sores]" (carer)

"Mostly I buy stuff from the shops. Medicated soaps and medicated bubble bath to treat my kids." (carer)

"I don't think they care before it starts being a problem. So when it starts being painful, when the kid starts to lose sleep over it, that's when they come, when things are past the initial stage of just being an easy fix with a small dressing. They wait till it's a big problem and there's more work involved in cleaning it up, preventing more, starting medication. It's sort of a vicious circle. They wait too long and then the treatment is bigger and more time-consuming. A lot of things could have started earlier and are preventable." (healthcare practitioner)

"They don't go get it fixed until it hurts them or until it causes problem. I knew a child a few years ago who had a skin infection that was left too long and then he lost his leg." (other service provider)

"I think they need to educate and understand skin infections more, otherwise they become normalised and people don't bother to go until it's really bad." (other service provider)

**FACTOR:**	**predisposing**	enabling	need
**LEVEL:**	**client**	provider	system

FACTOR:	predisposing	enabling	need
LEVEL:	client	provider	system

normalisation of skin infections

(discussed by C, HP, OSP)

"They also normalise it. They think 'oh it will get better' they will only take the kids to the clinic if it's really, really bad" (carer)

"I don't know what they see as a normal part of life in their skin problems. I know that most likely they're used to it. They're used to, "Mom had heaps of boils when she was young, so why should I start running to the clinic with my child when they have them, because they're just there anyway?"" (healthcare practitioner)

"It's not seen as being different. It's not seen as if there is anything wrong with it because all of the kids got sores. What's different between my kids having sores and everybody else?" (healthcare practitioner)

"Well I think because it's so common the mothers think 'oh it will go away'. They don't go in until its very, very bad." (other service provider)

"I think they need to educate and understand skin infections more, otherwise they become normalised and people don't bother to go until it's really bad." (other service provider)

FACTOR:	predisposing	enabling	need
LEVEL:	client	provider	system

**FACTOR:**	predisposing	enabling	**need**
**LEVEL:**	client	**provider**	system

clinic not providing sufficient health education

(discussed by C, HP, OSP)

"The healthcare in these communities is really poor because they don't teach us." (carer)

"If they don't educate they will all end up with that kidney and heart stuff." (carer)

"The nurses should be out there educating people on the skin stuff, not just treating." (carer)

"There needs to be… You know more treatment [education]. Say 'bathe him in this' or 'wipe him with this.' They don't, they just give medicine." (carer)

"I think we need to push to the AMS for more health promotion. And probably need someone to go visit the communities with pictures and the creams and what to do." (carer)

"So it's different ways of looking at it, but I think education towards the kids about their health needs to be developed and maintained in a different way. And I think the health care professions needs to step in and do it, and not rely on the teachers to do it, because the teachers have enough other things they need to educate the things in. I think by getting a new face in, explaining, showing, and then it might be one or two things the kids remember later on that might prevent one kid to have boils or scabies or something like that." (healthcare practitioner)

"The staff don't even tell them what to do with the medication or how to get rid of the sores. They need to educate them you know? […] The health clinic doesn't even give them soap or any stuff to rub on the sores they only give them medicine and no other treatment – no information on it either." (other service provider)

actively encourage child/carer to go to clinic

(discussed by OSP)

"If there's something that I don't like the look of, I just get the mom to take them to the clinic, or I do. The clinic's literally next door to us, so it's not an issue. It's not hard taking them. It's not a long trip or anything." (other service provider)

"We try and call the carer or the parent and say, "come and pick them up and take them". And nine times out of then they'll come and take them. Whether they take them to the clinic or take them home is a moot point. If it's really, really bad and we can't find anybody, then we make a decision to take them up or whatever. Sometimes you just can't get hold of the carer. You don't know where they are. Typically, we try and get the parent or the carer to take responsibility." (other service provider)

**FACTOR:**	**predisposing**	enabling	need
**LEVEL:**	**client**	provider	system

**FACTOR:**	predisposing	enabling	**need**
**LEVEL:**	client	provider	**system**

No quotes.

© 2020 by the authors. Submitted for possible open access publication under the terms and conditions of the Creative Commons Attribution (CC BY) license (http://creativecommons.org/licenses/by/4.0/).

**FACTOR:**	**predisposing**	enabling	need
**LEVEL:**	**client**	provider	system
